# Dissociation of Infectivity from Seeding Ability in Prions with Alternate Docking Mechanism

**DOI:** 10.1371/journal.ppat.1002128

**Published:** 2011-07-14

**Authors:** Michael B. Miller, James C. Geoghegan, Surachai Supattapone

**Affiliations:** 1 Department of Biochemistry, Dartmouth Medical School, Hanover, New Hampshire, United States of America; 2 Department of Medicine, Dartmouth Medical School, Hanover, New Hampshire, United States of America; University of Alberta, Canada

## Abstract

Previous studies identified two mammalian prion protein (PrP) polybasic domains that bind the disease-associated conformer PrP^Sc^, suggesting that these domains of cellular prion protein (PrP^C^) serve as docking sites for PrP^Sc^ during prion propagation. To examine the role of polybasic domains in the context of full-length PrP^C^, we used prion proteins lacking one or both polybasic domains expressed from Chinese hamster ovary (CHO) cells as substrates in serial protein misfolding cyclic amplification (sPMCA) reactions. After ∼5 rounds of sPMCA, PrP^Sc^ molecules lacking the central polybasic domain (ΔC) were formed. Surprisingly, in contrast to wild-type prions, ΔC-PrP^Sc^ prions could bind to and induce quantitative conversion of all the polybasic domain mutant substrates into PrP^Sc^ molecules. Remarkably, ΔC-PrP^Sc^ and other polybasic domain PrP^Sc^ molecules displayed diminished or absent biological infectivity relative to wild-type PrP^Sc^, despite their ability to seed sPMCA reactions of normal mouse brain homogenate. Thus, ΔC-PrP^Sc^ prions interact with PrP^C^ molecules through a novel interaction mechanism, yielding an expanded substrate range and highly efficient PrP^Sc^ propagation. Furthermore, polybasic domain deficient PrP^Sc^ molecules provide the first example of dissociation between normal brain homogenate sPMCA seeding ability from biological prion infectivity. These results suggest that the propagation of PrP^Sc^ molecules may not depend on a single stereotypic mechanism, but that normal PrP^C^/PrP^Sc^ interaction through polybasic domains may be required to generate prion infectivity.

## Author Summary

Prions are unconventional infectious agents that cause fatal diseases in humans and other animals. Previous studies have suggested that prion infectivity depends upon the ability of a sample to change the shape of a normal brain protein called the prion protein (PrP) into a disease-associated shape. Other studies have identified a pair of positively charged domains within the structure of PrP that appear to be important for the interaction between the normal and disease-associated shapes of the prion protein. In this report, we show that the shape of normal PrP can change into the disease-associated form through a novel mechanism that does not involve positively charged domains. However, it appears that interaction through the positively charged domains is required to produce infectious prions efficiently. Our results show for the first time that the ability to change the shape of normal PrP into its disease-associated state is not the sole determinant of prion infectivity.

## Introduction

Prions are infectious proteinaceous particles that cause fatal neurodegenerative diseases, including Creutzfeldt-Jakob disease (CJD), bovine spongiform encephalopathy (BSE), and chronic wasting disease (CWD). Prions contain PrP^Sc^, a protease-resistant detergent-insoluble β-sheet-rich conformer of the normal cellular protein PrP^C^ [1], [2]. PrP^Sc^ is an essential and possibly the sole component of infectious prions. Prion propagation and disease require the presence of PrP^C^, encoded by the host *Prnp* gene [3], [4], [5], [6].

Cell-free *in vitro* propagation systems have emerged as valuable tools to investigate PrP^Sc^ and prion propagation [7]. By serial protein misfolding cyclic amplification (sPMCA), prion infectivity can be propagated *in vitro* [8]. More rapid and less costly than the gold standard inoculation bioassay, sPMCA has been proposed as an *in vitro* method to detect prion infectivity [9]. Indeed, samples which seed robust sPMCA propagation have been previously associated with biological infectivity [8], [10]. However, it is unknown if PrP^Sc^ molecules that robustly seed PMCA propagation in wild type brain homogenate are always associated with appropriate levels of specific infectivity.

Recent studies have reconstituted infectious PrP^Sc^ propagation using purified PrP^C^ substrate and supplementary conversion cofactors, a set of minimal components that appear necessary for prion propagation [11], [12]. PrP^Sc^ appears to propagate by autocatalysis, binding PrP^C^ to induce conversion into a new PrP^Sc^ molecule [13]. However, the mechanisms of binding and conversion remain unclear.

Studies using motif-grafted antibodies or PrP-derived peptides identified two polybasic regions that bind strongly to PrP^Sc^ [14], [15], [16], suggesting that these PrP^C^ domains may serve as docking sites for PrP^Sc^. The N-terminal (N-PBD, 23–28) and central (C-PBD, 100–109) polybasic domains both fall in the N-terminal flexible region of PrP^C^, which is less ordered than the C-terminus [17], [18]. Antibodies directed at C-PBD can impede prion propagation in cultured cells and *in vivo* [19], [20], possibly by blocking a PrP^Sc^-binding site on PrP^C^. The polybasic domains may also be involved in interaction with lipid molecules during the conversion process [21] and may affect the structure of misfolded prion protein [22].

Mice overexpressing PrP transgenes lacking amino acids 23–88 show reduced prion susceptibility [23], but these studies did not examine N-PBD in isolation. Transgenic mice with deletions that include C-PBD display lethal neurologic illness, but this phenotype precluded inoculation experiments assessing prion susceptibility [24], [25], [26]. The specific role of these domains in the context of the entire PrP^C^ molecule has been studied in N2a cells co-expressing wild-type and mutant PrP^C^ [27], but biochemical examinations of binding interactions and of propagation with pure substrates are lacking.

In full-length PrP^C^, the relative importance of the N-terminal and central polybasic domains in binding PrP^Sc^ is unclear. Moreover, while much evidence points to a significant role for PrP^C^ polybasic domains, it is not known if PrP^Sc^-PrP^C^ interactions are universally stereotypic – whether all PrP^Sc^ molecules use the same PrP^C^ epitopes for interaction.

Using a combination of Chinese hamster ovary (CHO) cell expression, protein purification, and reconstitution sPMCA techniques, we engineered PrP^Sc^ molecules lacking polybasic domains. These novel PrP^Sc^ molecules, which propagate robustly *in vitro*, enabled us to assess whether PrP^C^ polybasic domains are universally required for PrP^Sc^ docking and propagation. PBD-deficient PrP^Sc^ molecules also provided us with a unique opportunity to dissect the relationship between the ability to seed formation of wild type PrP^Sc^ molecules *in vitro* and prion infectivity *in vivo*.

## Materials and Methods

### Mutagenesis and expression of recPrP-myc for binding

Sequence encoding mouse PrP-A 23–230 was amplified, using the following primers to add an N-terminal start codon and a C-terminal tag encoding amino acids 410–419 of human c-myc (NCBI accession number NP_002458.2): N: 5′- aaaaaacatatgaaaaagcggccaaagcctggagggt-3′, C: 5′-aaaactcgagtcattacagatcctcttctgagatgagtttttgttcggatcttctcccgtcgtaatag-3′. This fusion gene was inserted into pET22b(+) for bacterial expression. N-terminal (ΔN, 23–28) and central (ΔC, 100–109) polybasic domains were deleted by site-directed mutagenesis (GeneTailor, Invitrogen, Carlsbad, CA). From transfected *Escherichia coli* Rosetta cells, recombinant PrP was purified in a manner similar to that described by Wang *et al.* [12]. Cells from 500 mL induced (Overnight Express Autoinduction System, Novagen, EMD Chemicals, Gibbstown, NJ) overnight culture were pelleted at 8,000×*g* for 10 min.; lysed with 40 mL BugBuster, Lysonase (Novagen), and intermittent sonication over 20 min.; and inclusion bodies were prepared by two cycles of centrifugation (16,000×*g*, 15 min.) and 0.1× Bugbuster resuspension, followed by an additional 16,000×*g* 15 min. spin. Inclusion body pellets were solubilized in 8 mL 8 M guanidine hydrochloride, insoluble material removed by centrifugation (8,000×*g*, 10 min.), and protein added to 3.6 g Ni-NTA Hisbind Superflow resin (Novagen) pre-equilibrated in denaturing buffer (100 mM sodium phosphate, 10 mM Tris, 6 M Guanidine, 10 mM β-mercaptoethanol, pH 8.0). After 30 min. binding, resin was transferred to column support, and protein was refolded by a linear 12 hr. 125 mL gradient of denaturing to refolding buffer (100 mM sodium phosphate, 10 mM Tris, pH 8.0), followed by 60 mL wash at 1 mL/min. in refolding buffer. Protein was eluted from nickel affinity resin by 1 mL/min. 500 mM imidazole, 100 mM sodium phosphate pH 6.5; then dialyzed into 20 mM sodium phosphate pH 6.5 (3×2 L for 30 min.) and into water (3×2 L for 30 min., 1×4 L overnight). If observed, precipitate was removed by centrifugation at 100,000×*g* for 60 min. The nickel eluate was loaded on 2 mL pre-equilibrated (30 mL 10 mM sodium phosphate pH 6.5, 1 mL/min.) CM sepharose (Sigma, St. Louis, MO) at 0.5 mL/min., washed with 50 mL NaCl at 1 mL/min., and eluted with a gradient (300–650 mM NaCl, 0.5 mL/min.). Fractions containing >0.1 OD_280_ (>0.037 mg/mL PrP) [28] were pooled and dialyzed into water (2×4 L for 30 min., 1×4 L overnight), then stored at −70°C. Protein concentration was determined by bicinchoninic acid (BCA) assay.

### PrP^Sc^-PrP binding assays

RML scrapie-infected brain homogenate (5% BH in tris-buffered saline) or CHO ΔC-PrP^Sc^ (sPMCA round 16 product, therefore 10^16^-fold dilution of original wild-type scrapie seed) were vortexed for 15 sec., sonicated for 1 min. at 70% power (Misonix 4000 with Microplate Horn, Qsonica, Newtown, CT), and centrifuged at 500×*g* for 15 min. Normalized amounts of this clarified preparation (12.4 µL brain homogenate PrP^Sc^, 16.5 µL PrP^Sc^) were incubated with 3.5 µg purified *E. coli* recPrP-myc (wild-type or lacking polybasic domain) in 250 µL binding buffer (50 mM tris, 200 mM NaCl, 1% Triton X-100, 1% Tween-20, pH 7.5) for 1 hr. at 4°C with 10 r.p.m. end-over-end rotation. Concurrently, 16 µL 30 mg/mL Dynabeads Protein A (Invitrogen) was washed with 2×500 µL phosphate-buffered saline (PBS), collected by a Magnetic Particle Separator (PureBiotech, Middlesex, NJ), and incubated with 3.6 µg 9E10 anti-myc antibody (Santa Cruz Biotechnology, Santa Cruz, CA) in 250 µL binding buffer for 30 min. at room temp. with 10 r.p.m. end-over-end rotation. Next, the solution of PrP^Sc^ and PrP-myc was added to the bead-anti-myc complexes, and rotated for 1 hr. at 4°C. Following this incubation, beads were collected, and supernatant was aspirated. Beads were rinsed in 4×500 µL wash buffer (50 mM tris, 200 mM NaCl, 0.05% Tween 20), and analyzed for bound PrP^Sc^. Signal intensities were quantified by densitometry with ImageGauge V4.22 (Fujifilm) in quant mode.

### Expression and preparation of PrP from Chinese hamster ovary (CHO) cells

Sequences encoding wild-type and polybasic domain deletion mutant (ΔN = Δ23–28; ΔC-PBD = Δ100–109; ΔΔ-PBD = Δ23–28 Δ100–109) PrP were inserted in pcDNA5/FRT plasmids. These were used to express PrP from CHO cells, which was prepared as described previously [29]. For hamster experiments, homologous deletion mutants were used (Δ23–28, Δ101–110, or ΔΔ).

### Reconstituted serial protein misfolding cyclic amplification (sPMCA) with CHO-expressed PrP

Reactions were prepared and carried out as described by Geoghegan *et al.* [29]. Briefly, CHO-expressed mouse PrP (wild-type or polybasic domain deletion mutant) was mixed with *Prnp^0/0^* (Zurich) brain homogenate (2.5% final concentration). Ninety microliters of this reconstituted substrate was mixed with 10 µL seed. Reactions were initially seeded with 0.1% scrapie-infected brain homogenate (mouse strain RML) in PBS, with 10 µL of product used to seed the subsequent round. ΔC-PrP^Sc^ seed was produced by 16 rounds of sPMCA (10^16^-fold dilution of original wild-type scrapie seed), containing <1 original PrP^Sc^ molecule [8]. Unseeded reactions were given 10 µL PBS as initial seed. For reactions lacking cofactor, buffer (PBS 1% Triton X-100) replaced *Prnp^0/0^* brain homogenate. Each PMCA round consisted of incubation at 37°C for 24 hr. with 20 sec. microplate horn sonication at 85% power every 30 min. One set of seeded samples was not subjected to PMCA (round 0), while another set was not subjected to protease digestion (−PK) to observe input PrP. For hamster sPMCA experiments, or Sc237 strain was used, CHO HaPrP was supplemented with 20 µg/mL synthetic poly(A) RNA (Sigma, St. Louis, MO), and PrP^Sc^ was detected by immunoblot after 50 µg/mL digestion for 60 min.

### sPMCA with brain homogenate

Brain homogenate sPMCA experiments were adapted from Castilla *et al.* [30]. 90 µL 10% CD-1 mouse brain (Biochemed, Winchester, VA) homogenate was prepared in PBS, 1% Triton X-100, 5 mM EDTA, Roche Complete mini protease inhibitor, then seeded with PrP^Sc^ (10 µL of round 16 product). One round of PMCA consisted of 30 sec. 90% power microplate horn sonication pulses every 30 min. for 24 hr. at 37°C. 10 µL of each reaction product was transferred to fresh brain homogenate for the following round.

### PrP^Sc^ detection

Mouse PrP^Sc^ was detected by digestion with 25 µg/mL proteinase K (Roche, Indianapolis, IN) for 30 min. at 37°C and 750 r.p.m. shaking, polyacrylamide gel electrophoresis (PAGE), transfer to polyvinylidene fluoride (PVDF), and Western detection with anti-PrP and horseradish peroxidase-conjugated anti-mouse sheep IgG (GE Healthcare, Piscataway, NJ). Signals were detected by enhanced chemiluminescence (ECL) (SuperSignal West Femto Substrate, Pierce, Rockford, IL) and visualized by a Fuji (Fujifilm) LAS-3000 chemiluminescence documentation system. Hamster PrP^Sc^ was detected with 50 µg/mL proteinase K for 60 min. Experiments detecting only wild-type PrP used 6D11 anti-PrP antibody. Because the central polybasic domain (C-PBD) forms part of the 6D11 epitope, and therefore ΔC PrP is not detected by 6D11 (data not shown), reconstituted sPMCA experiments utilized anti-PrP mAb 27/33.

### Biological infectivity assay

**Figure 1:**
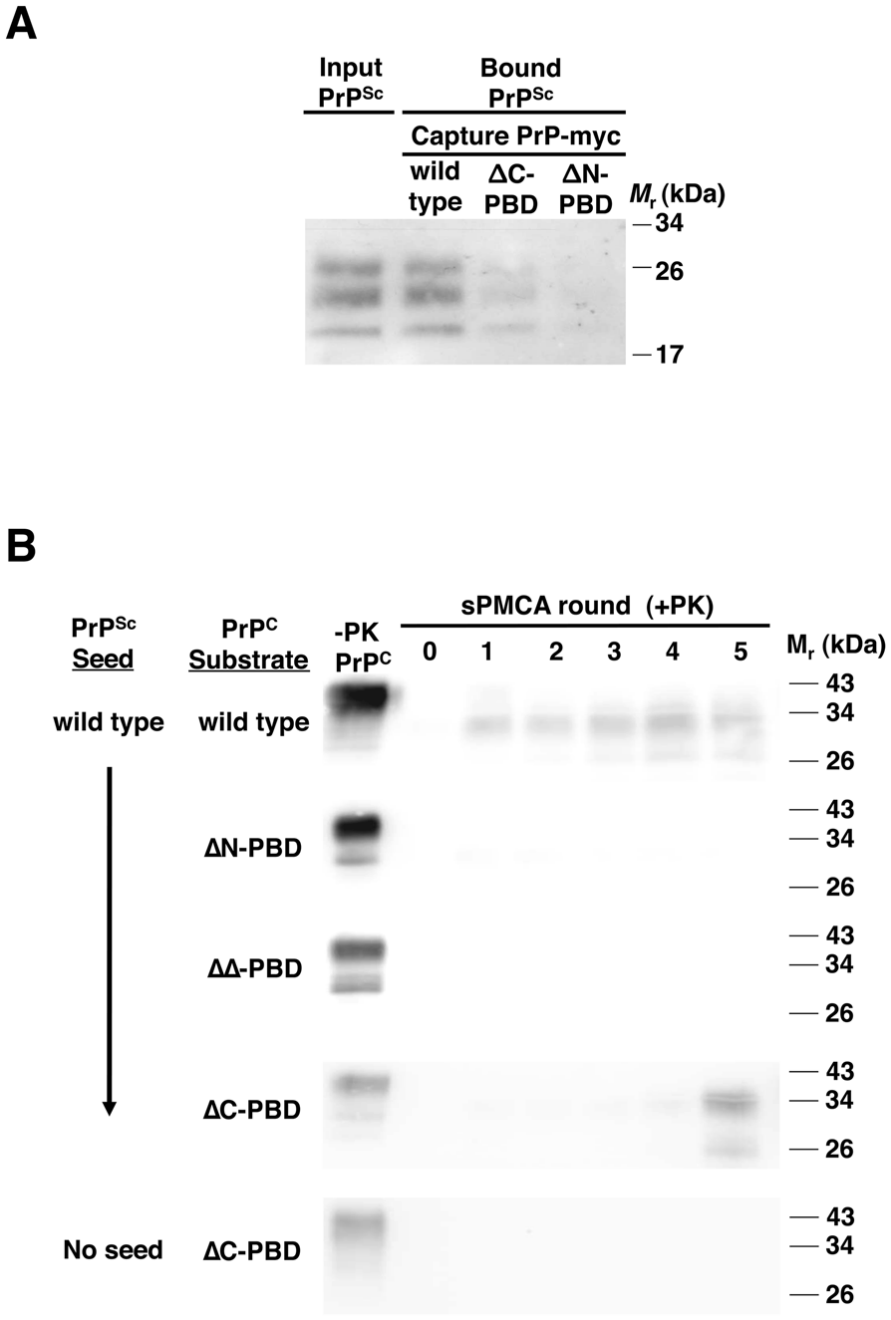
Interaction of wild-type PrP^Sc^ with mutant PrP molecules. (*A*) Binding of PrP^Sc^ to PrP. RML scrapie-infected mouse brain homogenate was incubated with myc-tagged PrP of wild-type sequence or lacking the central (ΔC-PBD: Δ100–109) or N-terminal (ΔN-PBD: Δ23–28) polybasic domain. Bound PrP^Sc^ was captured with 9E10 anti-myc antibody on magnetic protein A Dynabeads, and detected by 25 µg/mL proteinase K digestion and anti-PrP (6D11) immunoblot. (*B*) Propagation of PrP^Sc^. RML scrapie-infected mouse brain homogenate was propagated by serial protein misfolding cyclic amplification (sPMCA) for five rounds with wild-type or polybasic deletion mutant PrP^C^ prepared from Chinese hamster ovary (CHO) cells. Reactions were supplemented with *Prnp^0/0^* mouse brain homogenate. In addition to scrapie-seeded reactions, an unseeded reaction was performed with ΔC-PBD PrP substrate. One sample of each reaction was not subjected to protease digestion (−PK). All others were subjected to limited proteolysis with 25 µg/mL proteinase K. PrP was detected by immunoblot (anti-PrP 27/33).

**Figure 2:**
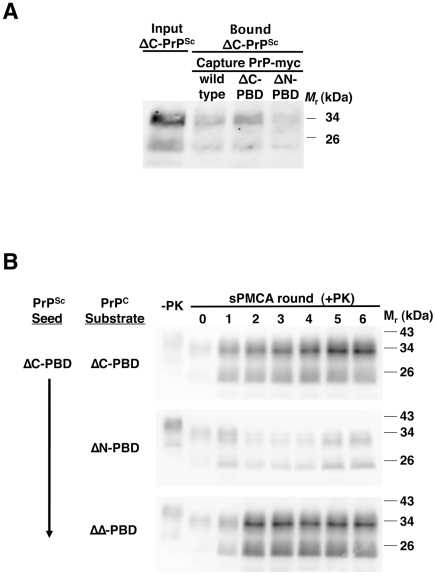
Interaction of ΔC-PBD PrP^Sc^ with mutant PrP molecules. (*A*) Binding of ΔC-PBD PrP^Sc^ to PrP. ΔC-PBD PrP^Sc^ was incubated with wild-type or polybasic mutant (ΔC-PBD, ΔN-PBD) myc-tagged PrP. Bound PrP^Sc^ was captured with 9E10 anti-myc antibody on magnetic protein A Dynabeads, and detected by 25 µg/mL proteinase K digestion and anti-PrP (27/33) immunoblot. (*B*) Propagation of ΔC-PBD PrP^Sc^. ΔC-PBD PrP^Sc^ was propagated by sPMCA with polybasic deletion mutant PrP^C^ prepared from Chinese hamster ovary (CHO) cells. Reactions were supplemented with *Prnp^0/0^* mouse brain homogenate. One sample of each reaction was not subjected to protease digestion (−PK), loading ¼ of volume. All others were subjected to limited proteolysis by 25 µg/mL proteinase K digestion. PrP was detected by immunoblot (anti-PrP 27/33).

RML PrP^Sc^, propagated with CHO wild-type or ΔC- PrP^C^ *in vitro* by sPMCA (Figures 1B, S4) for at least 14 rounds, was diluted 1∶10 in diluent (PBS+1 mg/mL BSA). ΔN- and ΔΔ-PrP^Sc^ molecules, propagated from ΔC-PrP^Sc^ (Figure 2B), were prepared in the same manner. By serial dilution, each inoculum contained 10^−15^ of the original seed, equivalent to less than one original PrP^Sc^ molecule [8]. PrP^Sc^ preparations (30 µL) were injected intracerebrally into female CD-1 mice aged 6 weeks (Charles River Laboratories, Wilmington, MA). For two samples (ΔC-PrP^Sc^ and ΔΔ-PrP^Sc^), end-point dilution bioassays were performed, in which 10-fold serial dilutions were inoculated to measure the amount of prion infectivity present in the original sample. As described previously [11], animals were monitored daily for clinical signs of neurological dysfunction over the standard one-year observation period. Animals showing terminal scrapie were sacrificed, and their brains were analyzed for PrP^Sc^ (by 25 µg/mL protease digestion and immunoblot) and for spongiform degeneration (by hematoxylin & eosin histology) [11]. Random asymptomatic animals were sacrificed after one year, and their brains were likewise analyzed for PrP^Sc^ and spongiform degeneration.

### Ethics statement

All animals were handled in strict accordance with good animal practice, as defined by the Guide for the Care and Use of Laboratory Animals of the National Institutes of Health. The Dartmouth College Institutional Animal Care and Use Committee approved the animal work (assurance number A3259-01). Inoculations were performed under isoflurane anesthesia, and all efforts were made to minimize suffering.

## Results

### Interaction and propagation of wild-type PrP^Sc^ with PrP molecules lacking polybasic domains

Studies using fragments of PrP identified N-terminal (23–28) and central (100–109 in mouse) polybasic domains as potential binding sites for PrP^Sc^ [14], [16]. To investigate the role of these domains in the context of full-length PrP^C^, we developed a novel magnet-based assay to examine direct interaction between PrP^Sc^ and PrP^C^ molecules with or without deletions of these polybasic regions. In this assay, PrP^Sc^ was incubated with purified recombinant myc-tagged PrP, and then adherent molecules were pulled down by α-myc antibodies attached to Protein A magnetic beads. Substitution of a short myc epitope in place of the C-terminal glycophosphatidylinositol anchor of PrP^C^ permitted specific capture. The assay specifically assesses PrP^Sc^ binding to PrP (Figure S1 in Supporting Information S1), and indicates that purified PrP^Sc^ binds to purified PrP (Figure S2 in Supporting Information S1). Next, we compared binding of PrP^Sc^ to wild-type and mutant PrP substrates. In contrast to quantitative RML PrP^Sc^ binding by wild-type PrP (∼100% by densitometry), PrP lacking the central polybasic domain (ΔC) bound significantly less PrP^Sc^ (∼50%) and N-terminal polybasic deletion mutant PrP (ΔN) bound still less PrP^Sc^ (∼20%) (Figure 1A). Purified PrP^Sc^ also adhered to wild-type PrP more strongly than to polybasic mutant PrP (Figure S2 in Supporting Information S1). These results suggest that both PrP polybasic domains contribute to PrP^Sc^ binding.

To test the function of PrP^C^ polybasic domains in prion propagation, we performed sPMCA experiments with wild type and mutant PrP^C^ substrate. These PrP^C^ molecules, prepared from CHO cells transfected with wild-type or mutant *Prnp* DNA, were detergent-soluble (Figure S3 in Supporting Information S1) and membrane-anchored (Figure S4 in Supporting Information S1), indicative of proper folding and intracellular trafficking. In reconstituted, three-round sPMCA experiments with PrP^C^ and *Prnp^0/0^* brain homogenate, PrP^Sc^ propagated efficiently, converting the PrP^C^ to protease-resistant autocatalytic PrP^Sc^ molecules (Figure 1B and S5 in Supporting Information S1). In contrast, PrP^C^ molecules missing one (ΔN, ΔC) or both (ΔΔ) polybasic domains did not support efficient propagation, indicating that PrP^C^ requires these domains to facilitate PrP^Sc^ propagation. This result was also found with Sc237 hamster prions, as we observed no propagation of protease-resistant PrP with polybasic mutant hamster PrP^C^ (Figure S5 in Supporting Information S1).

When we examined propagation in polybasic mutant substrates beyond three rounds, surprisingly, one mutant (ΔC) reproducibly produced PrP^Sc^ molecules, typically between rounds 3–5 (Figure 1B). Further, these ΔC-PrP^Sc^ molecules propagated robustly and indefinitely (Figure S6 in Supporting Information S1), indicating capability of PrP^C^ to support propagation despite absence of the central polybasic domain. Indeed, ΔC-PrP conversion was extremely efficient, with ∼100% of substrate converted to PrP^Sc^, outpacing even wild-type PrP conversion ratios (∼20% of substrate). ΔC-PrP^Sc^ molecules appear to derive from the original wild-type PrP^Sc^ seed, as unseeded reactions did not generate PrP^Sc^ (Figure 1B).

### Interaction range of ΔC-PrP^Sc^ molecules

The unexpected generation of ΔC-PrP^Sc^ molecules led us to conduct a series of experiments to assess the role of polybasic domains in ΔC-PrP^Sc^ propagation. Using the magnetic myc-capture assay, we tested binding to various PrP substrates. In contrast to wild-type PrP^Sc^, ΔC-PrP^Sc^ reproducibly bound more strongly to ΔC-PrP (∼50% of input) than to wild type PrP (∼35% of input) (Figure 2A). Furthermore, ΔC-PrP^Sc^ bound to ΔN-PrP (∼30% of input), suggesting a significantly different interaction mechanism than wild-type PrP^Sc^. Interestingly, wild-type PrP^Sc^-PrP^C^ binding appeared stronger than ΔC-PBD mutant PrP^Sc^-PrP^C^ interactions.

We next examined ΔC-PrP^Sc^ propagation with other polybasic mutant substrates. Fitting with its binding behavior, ΔC-PrP^Sc^ propagated successfully in ΔN-PrP^C^ (Figure 2B). Moreover, the ΔC-PrP^Sc^ seed also propagated in ΔΔ-PrP^C^ with robust conversion, indicating that presence of a polybasic domain is not an absolute requirement for PrP^C^ to convert to a protease-resistant autocatalytic form. Indeed, all three types of ΔPBD-PrP^Sc^ molecules propagated robustly, with stringent proteinase K digestion (25 µg/mL) revealing a protease-resistant core with a ∼7 kDa molecular weight shift.

### Cofactor dependence and seeding specificity of PrP^Sc^ molecules lacking polybasic domains

**Figure 3:**
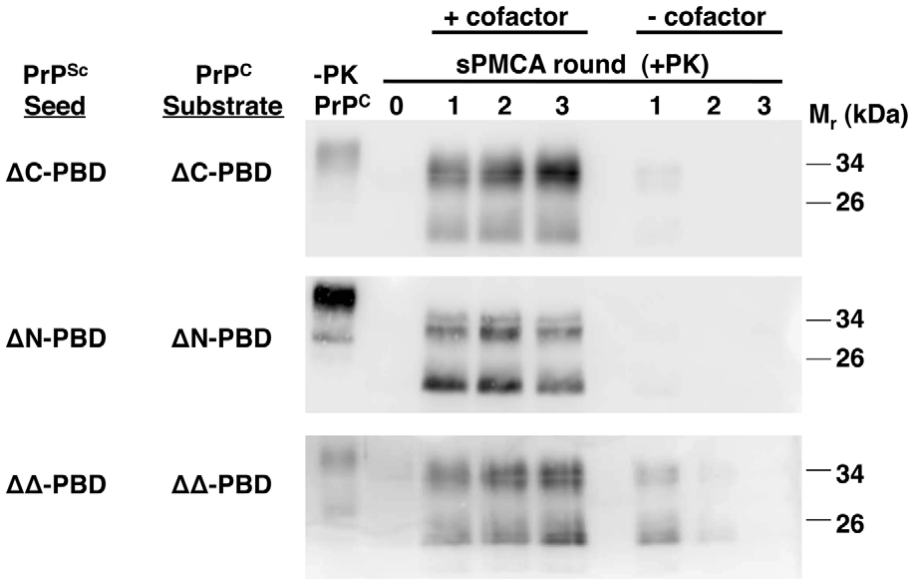
Effect of accessory cofactors on the propagation of ΔPBD PrP^Sc^ molecules. *In vitro*-generated ΔC-PBD, ΔN-PBD, and ΔΔ-PBD PrP^Sc^ molecules were propagated by sPMCA with autologous PrP^C^ prepared from Chinese hamster ovary (CHO) cells. One set of reactions was supplemented with *Prnp^0/0^* mouse brain homogenate (+cofactor), while a second set received only buffer (−cofactor). One sample of each reaction was not subjected to protease digestion (−PK), while all others were digested with 25 µg/mL proteinase K. –PK reactions for ΔC-PBD and ΔΔ-PBD were loaded with ¼ volume. PrP was detected by immunoblot (anti-PrP 27/33).

Infectious wild-type PrP^Sc^ molecules depend on accessory cofactor molecules for propagation [11], [12], [31]. We tested whether polybasic domain mutant PrP^Sc^ molecules exhibit this characteristic by performing parallel sPMCA reactions, omitting the supplemental *Prnp^0/0^* brain homogenate from one set (Figure 3). All three PrP^Sc^ mutants (ΔC, ΔN, and ΔΔ) failed to propagate in the absence of *Prnp^0/0^* brain homogenate, pointing to a cofactor-dependent propagation mechanism. The signals seen in initial rounds of cofactor-negative propagation are likely due to the presence of residual *Prnp^0/0^* brain homogenate carried over from the PrP^Sc^ seed mixture. By the third round of sPMCA in cofactor-free substrate, no PrP^Sc^ molecules were detected.

**Figure 4:**
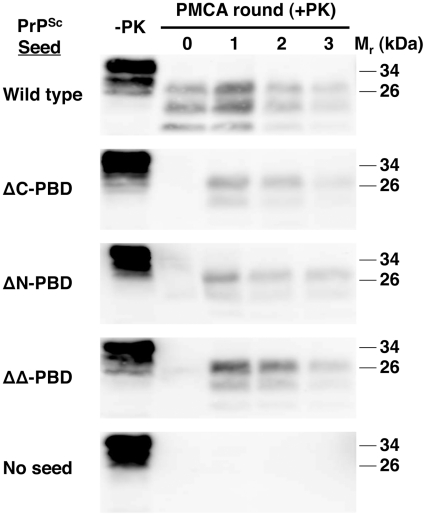
ΔPBD PrP^Sc^ molecules seeding wild-type brain homogenate sPMCA reactions. *In vitro*-generated ΔC-PBD, ΔN-PBD, and ΔΔ-PBD PrP^Sc^ molecules were propagated by sPMCA for three rounds with wild-type mouse brain homogenate, containing wild-type PrP^C^ substrate. Control reactions seeded with wild-type native RML prions and unseeded reactions were also tested. One sample of each reaction was not subjected to protease digestion (−PK), while all others were digested with 25 µg/mL proteinase K. PrP was detected by immunoblot (anti-PrP 6D11).

Infectious PrP^Sc^ seeds propagation in wild-type brain homogenate [32], [33] to the extent that successful sPMCA propagation is considered potentially diagnostic for prion infectivity [9]. We tested if ΔPBD-PrP^Sc^ molecules could seed propagation in wild-type brain homogenate (Figure 4). Like wild-type PrP^Sc^, all three PrP^Sc^ polybasic mutants (ΔC, ΔN, and ΔΔ) seeded serial propagation with success. Each of these reactions displayed positive conversion signals in round 1, suggesting a high amount of prion infectivity [34]. Interestingly, the PrP^Sc^ molecules that formed were predominantly diglycosylated, whereas PrP^Sc^ molecules formed from wild-type seeds displayed the three glycoforms in equivalent amounts. It has been shown that the ability to produce different PrPSc glycoforms is not due to preferential interaction, but rather inherent conformation [35]. This study found that mouse RML prions only require unglycosylated PrP^C^ for propagation, and that glycosylated forms of PrP^Sc^ can be made in its presence. This suggests that the observed glycosylation pattern described here may be caused by a different fold in polybasic mutant PrP^Sc^.

Thus, all three PBD mutant PrP^Sc^ molecules share the hallmark biochemical characteristics of infectious wild-type PrP^Sc^ molecules [11]: (1) a highly protease-resistant core, (2) cofactor-dependent propagation, and (3) the ability to seed wild-type brain homogenate sPMCA reactions.

### Infectivity of polybasic mutant PrP^Sc^ molecules

**Figure 5:**
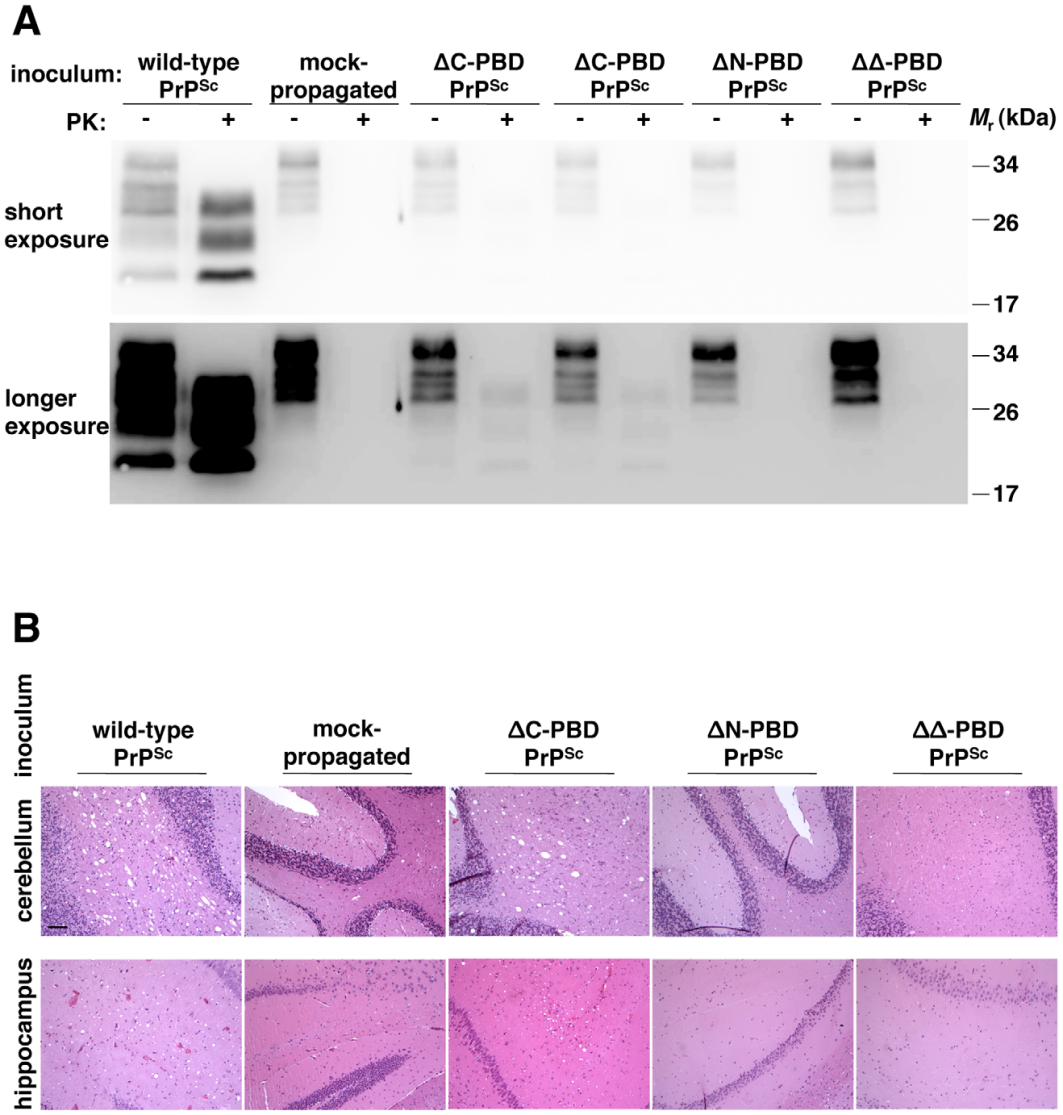
Biochemical and neuropathological analysis of mice inoculated with *in vitro*-generated PrP^Sc^ molecules. Brains were dissected from wild-type mice showing terminal scrapie signs (PrP^Sc^ and ΔC-PBD PrP^Sc^ inocula) or similarly aged mice not displaying scrapie signs (mock-propagated, ΔN-PBD PrP^Sc^, and ΔΔ-PBD PrP^Sc^ inocula). (*A*) Equivalent amounts of 10% brain homogenate were treated with buffer (−PK) or 25 µg/mL proteinase K (+PK to show PrP^Sc^) and detected by anti-PrP (6D11) immunoblot. A greater exposure of the same immunoblot is displayed below, to illustrate samples containing low amounts of PrP^Sc^. (*B*) Neuropathology of cerebellum and hippocampus. Brain sections were stained with hematoxylin and eosin (H&E). The black bar denotes 100 µm.

We tested whether mutant PrP^Sc^ molecules, generated *in vitro* from CHO-expressed PrP, are infectious to animals. Following sPMCA propagation sufficient to dilute out original seeds, wild-type and mutant (ΔC, ΔN, and ΔΔ) PrP^Sc^ molecules were inoculated intracerebrally into wild-type mice. Wild-type CHO PrP^Sc^ contains significant infectivity, causing scrapie disease in all animals with an incubation period comparable to RML prions propagated *in vitro* in brain homogenate [36] (Table 1). Affected animals displayed accumulation of protease-resistant prion protein and significant histopathological spongiform degeneration (Figure 5), as we previously reported for hamster prions propagated *in vitro* in wild-type CHO-expressed hamster PrP [29]. Animals inoculated with wild-type CHO PrP^Sc^ also showed robust levels of protease-resistant PrP^Sc^ on Western blot.

**Table 1:** Biological infectivity assay of *in vitro*-generated autocatalytic PrP^Sc^ molecules.

			Incubation period (days)		
Inoculum	Catalytic PrP^Sc^	Dilution	dScrapie killed	eKilled, not scrapie	fUnspecified death
Wild-type PrP^Sc^	+	10^−1^	162,162,193,203		
ΔC-PBD PrP^Sc^	+	10^−1^	453,460,469	371	350,418
		10^−2^	461,547		317,397
		10^−3^	611	398	388,507
		10^−4^	547	275,505	
		10^−5^			245,421,448,587
		10^−6^		426,635,635	
		10^−7^		635,635,635	427
a ΔN-PBD PrP^Sc^	+	10^−1^	564	455	360,394,405,421,480,511
a ΔΔ-PBD PrP^Sc^	+	10^−1^		457,477	239,294,347,450,466,490
		10^−2^		393	371,541,576
		10^−3^		233,478	464,595
		10^−4^		581	456,556
		10^−5^		602,602	428,498
		10^−6^		337,366,548	450
		10^−7^		602,602	490,590
b No PrP	−	10^−1^		427,635	576
c ΔC-PBD PrP^C^	−	10^−1^		635,635	476
c ΔN-PBD PrP^C^	−	10^−1^		602,602,602,602	
c ΔΔ-PBD PrP^C^	−	10^−1^		602,602,602	498,543,548

RML prions were propagated *in vitro* by sPMCA with purified CHO-expressed PrP^C^+*Prnp^0/0^* brain homogenate.

a Generated by *in vitro* propagation of ΔC-PBD PrP^Sc^ (Figure 2).

b Propagation in mock-purified untransfected CHO lysate+*Prnp^0/0^* brain homogenate.

c Unseeded PMCA reactions.

d Upon observation of clinical signs, scrapie illness was confirmed by Western blot for protease-resistant PrP and histopathology for spongiform degeneration.

e These animals showed no clinical signs of scrapie illness, and upon death or sacrifice were found to be negative biochemically for protease-resistant PrP and pathologically for spongiform degeneration. After >600 days post-inoculation, all remaining animals were sacrificed, analyzed for PrP^Sc^, and included in this table.

f These animals showed no clinical signs of scrapie illness, but tissue was not obtained due to sacrifice for other condition (such as dermatitis) or sudden death.

In contrast to wild-type, polybasic mutant PrP^Sc^ molecules show diminished *in vivo* infectivity. ΔC-PrP^Sc^ caused scrapie disease only in a fraction of animals, including those receiving the most concentrated inocula, with an incubation time 2.5-fold greater than animals inoculated with wild-type PrP^Sc^. ΔC-PrP^Sc^-inoculated diseased animals showed mild vacuolation (Figures 5B and S7 in Supporting Information S1) and displayed low levels of PrP^Sc^ on Western blot, with diglycosylated molecules most abundant. Such a glycoform ratio contrasts with the even distribution of glycoforms in animals inoculated with wild-type PrP^Sc^ (Figures 5A and S8 in Supporting Information S1), also seen with *in vitro* propagation (Figure 4). Both wild-type and ΔC-PrP^Sc^ induced scrapie illness characterized neurologically by lethargy and ataxia. ΔN-PrP^Sc^ molecules induced scrapie illness in a single animal, following a 564-day incubation period (Figure S9 in Supporting Information S1), with other such inoculated animals showing no scrapie illness (Figure 5). Due to the sudden nature of some deaths, we did not obtain tissue from all animals, but brain samples from many non-scrapie deaths were obtained as indicated in Table 1. ΔΔ-PrP^Sc^ molecules did not induce scrapie illness, as gauged by clinical observation and lack of protease-resistant PrP or histopathological change in animals sacrificed over one year after inoculation (Figure 5). Thus, while polybasic deletion mutant PrP^Sc^ molecules recapitulated *in vitro* many characteristics of infectious PrP^Sc^ molecules, they displayed low specific infectivity in wild-type mice.

## Discussion

In this study, we have engineered polybasic domain deficient PrP^Sc^ molecules that efficiently interact with and catalyze the conversion of an expanded range of PrP^C^ substrates, including native PrP^C^ in wild-type mouse brain homogenate. Remarkably, despite possessing the biochemical characteristics and *in vitro* seeding activity of wild-type prions, PBD-deficient PrP^Sc^ molecules possessed little or no *in vivo* infectivity.

### Requirement of PrP^C^ polybasic domains for propagation of wild-type PrP^Sc^

Studies of PrP fragments have indicated that polybasic domains can interact with PrP^Sc^ [14], [16]. To investigate both prion binding and conversion in the context of the entire PrP molecule, we designed mutant prion proteins lacking one or both polybasic domains, which were detergent-soluble and properly trafficked, as determined by PI-PLC release. Using a novel magnetic capture assay, we found that both PrP^C^ polybasic domains were required for optimal PrP^Sc^ binding. In reconstituted sPMCA experiments with PrP expressed and prepared from CHO cells, deletion of either or both polybasic domains of substrate PrP^C^ prevented efficient propagation of wild-type PrP^Sc^. As mutants lacking either domain did not efficiently bind or propagate wild-type PrP^Sc^, both PrP^C^ polybasic domains appear required to contact PrP^Sc^ for efficient propagation of wild-type prions.

Work using N2a cells co-expressing wild-type and mutant PrP suggested that the N-terminal polybasic domain may not play a role in conversion [27]. However, the presence of endogenous wild-type PrP^C^ may have provided conditions that facilitated conversion of the weaker-binding mutant PrP substrate. The results presented in this paper, using mutant substrate alone to test binding and propagation, suggest that the PrP^C^ N-terminal polybasic domain indeed participates in wild-type PrP^Sc^ binding and propagation.

### Delayed conversion of PrP lacking polybasic domains

When sPMCA reactions seeded with wild-type prions were carried out to 3–5 rounds, ΔC-PrP^C^ substrate molecules converted to a form resistant to protease digestion under stringent conditions (25 µg/mL proteinase K at 37°C). After protease digestion, this form displayed a classical shift in molecular weight of ∼7 kDa. This form was also autocatalytic, continuing to propagate robustly (∼100% conversion) and indefinitely in ΔC-PrP^C^ substrate. In seeking to understand the surprising kinetics of the initial conversion event, which occurred after four rounds and 10^−4^ dilution of the original seed, we found that unseeded control reactions did not generate the mutant PrP^Sc^ product. Thus, ΔC-PrP^Sc^ did not arise *de novo* [11], but may have emerged from amplification of a few molecules of a minority conformation present in the original PrP^Sc^ seed. Such an event is consistent with recent reports of multiple prion strains emerging from a single “pure” source [37], [38], and 4–5 serial amplification rounds are required to detect PrP^Sc^ conformers at very low concentrations [34].

### Expanded substrate range and increased converting efficiency of ΔC-PrP^Sc^

**Figure 6:**
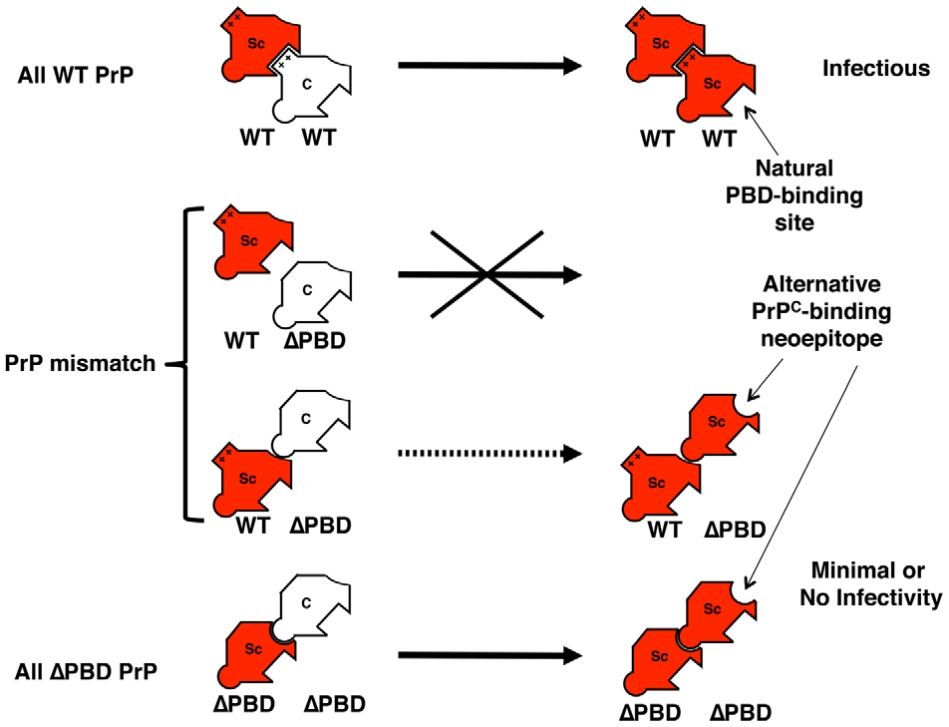
Model of PrP replicative interaction mechanisms. This diagram summarizes a proposed model for the interaction, propagation, and infectivity behavior of wild-type and polybasic deletion mutant PrP molecules. Wild-type PrP^Sc^ seed binds and propagates efficiently with autologous PrP^C^ substrate, using PrP^C^ polybasic domains (represented by rectangular protrusion) for docking. However, if PrP^C^ lacks one or both polybasic domains, PrP^Sc^ binds less well and exhibits impaired propagation. If PrP^Sc^ molecules lacking polybasic domains can be formed, they can bind and propagate efficiently with an expanded range of PrP^C^ substrates. ΔPBD-PrP^Sc^ may propagate by a different mechanism than wild-type PrP^Sc^, utilizing different residues (symbolized by round protrusion) of PrP^C^ for binding. A neoepitope (round depression) may be exposed in the polybasic mutant PrP^Sc^ molecules. Legend: Sc = PrP^Sc^; C = PrP^C^; WT = wild-type. ΔPBD = deletion in polybasic domain; ++ = polybasic domain.

Once formed, the novel ΔC-PrP^Sc^ molecules bound to and propagated in ΔN-PrP^C^ and ΔΔ-PrP^C^ substrates, forming new PrP^Sc^ molecules lacking either one or both polybasic domains. Thus, in contrast to wild-type PrP^Sc^, ΔC-PrP^Sc^ is universally catalytic in polybasic mutant PrP^C^, exhibiting an expanded substrate range that points to an alternative docking mechanism during propagation. Each of the ΔPBD PrP^Sc^ species propagated robustly, including even the mutant lacking both polybasic domains (ΔΔ). While wild-type PrP^Sc^ propagates by binding to substrate polybasic domains, ΔPBD mutant PrP^Sc^ molecules appear to utilize a different mechanism to bind PrP^C^, as outlined by the model in Figure 6. This indicates that prion docking, and perhaps other events in conversion, do not necessarily follow a rigidly conserved mechanism. In contrast, self-replicating fungal prions appear to utilize a stereotypic mechanism requiring glutamine/asparagine repeats [39].

The expanded interaction range of mutant PrP^Sc^ molecules could be explained by the exposure of a neoepitope on mutant PrP^Sc^ (Figure 6). If ΔPBD mutant PrP^Sc^ molecules propagate from a minority constituent conformer within wild-type PrP^Sc^, the putative neoepitope may also be present on these minority seed molecules. Mechanisms of PrP^Sc^ docking may also vary between different natural prion strains. Further studies are required to assess binding-site heterogeneity of PrP^Sc^-PrP^C^ interactions, including identification of specific docking sites used by ΔΔ-PBD molecules.

We also observed that ΔC-PrP^Sc^ molecules, once formed, catalyzed nearly complete conversion of autologous substrate, ∼5-fold greater than wild-type PrP^Sc^ autocatalysis. Thus, ΔC-PrP^Sc^ molecules show robust catalytic activity in terms of both substrate range and prion protein converting efficiency.

### Polybasic deficient PrP^Sc^ molecules resemble wild-type PrP^Sc^ *in vitro*

Our biochemical analysis revealed that ΔPBD PrP^Sc^ mutants possess all of the biochemical hallmarks that characterize wild-type infectious prions. All three ΔPBD PrP^Sc^ mutants (ΔC, ΔN, and ΔΔ) drive conversion of PrP^C^ in wild-type brain homogenate sPMCA experiments, a model of the conversion event in prion pathogenesis [8]. Furthermore, ΔPBD PrP^Sc^ molecules are resistant to stringent protease digestion, demonstrating a post-digestion molecular weight shift that is characteristic of PrP^Sc^. ΔPBD PrP^Sc^ molecules are also autocatalytic, propagating indefinitely in autologous PrP^C^ substrate. The propagation of ΔPBD PrP^Sc^ requires an accessory cofactor provided by *Prnp^0/0^* brain homogenate. Thus, unlike some fungal protein conformations, which have been shown to propagate *in vitro* with only the alternatively folded substrate protein [40], [41], [42], [43], ΔPBD PrP^Sc^ behaves like infectious mammalian prions in requiring a supplementary cofactor for propagation [11], [12], [31], [44], [45].

### Dissociation of brain homogenate sPMCA seeding activity from *in vivo* infectivity

PrP^Sc^ table-1-captionmolecules generated from CHO-expressed wild-type PrP^C^ induced scrapie illness in wild-type mice with a 100% attack rate (scrapie incubation time = 180±11 days). In contrast, despite exhibiting the *in vitro* hallmarks of wild-type infectious PrP^Sc^ as described above, mutant ΔC-PrP^Sc^ and ΔN-PrP^Sc^ molecules showed low infectivity *in vivo* (≤3000 LD_50_ units/mL and ∼300 LD_50_ units/mL, respectively), and ΔΔ-PrP^Sc^ showed no infectivity. Some inoculum-host PrP sequence N-terminal differences can mildly prolong scrapie incubation time, but these did not alter attack rates [46], [47], and much larger N-terminal PrP^Sc^ deletions than the ΔN-PrP^Sc^ that we report here did not significantly affect incubation time or attack rate in wild-type animals [23]. For example, protease-digested PrP^Sc^ yields PrP27-30, which is highly infectious to wild-type animals despite lacking amino acids 23–88 [48], [49]. Given that ΔPBD PrP^Sc^ molecules drive robust propagation *in vitro* in wild-type brain homogenate sPMCA, their diminished biological infectivity was highly unexpected, and represents dissociation between *in vitro* catalysis and *in vivo* infectivity, most notably for ΔN-PBD and ΔΔ-PBD PrP^Sc^.

To our knowledge, this report is the first demonstration of absent or minimal infectivity in samples that successfully seed propagation of wild-type brain homogenate during sPMCA, suggesting that the normal route of PrP^C^/PrP^Sc^ interaction through polybasic domains may be required for generating infectious prions. Why might appropriate PBD-mediated interaction be required for infectivity? One possibility is that PBD-deficient PrP^Sc^ molecules may be more susceptible to existing host mechanisms for prion clearance [50], [51], [52], [53], perhaps by exposure of a neoepitope on PrP^Sc^ that serves as a clearance signal. If this explanation were correct, then all three PBD-deficient PrP^Sc^ molecules must be preferential targets for the clearance mechanism.

A more plausible explanation is that the non-PBD mediated interaction mechanisms used by ΔPBD-PrP^Sc^ molecules to propagate *in vitro* lead to the production of alternative PrP^Sc^ conformations that are intrinsically non-infectious or have reduced infectivity. Consistent with this explanation, we observed different PrP^Sc^ glycosylation patterns in animals inoculated with ΔC-PrP^Sc^ compared to animals inoculated with wild-type PrP^Sc^. Such a distinction could be caused by an altered ability of mutant PrP^Sc^ to interact with non-PrP host molecules during *in vivo* propagation. For example, the *in vitro* detergent micelle environment differs from the membrane environments where propagation occurs *in vivo*. On the other hand, high titers of strain-preserved prion infectivity are propagated *in vitro* in detergent micelles [8], [11], [32], suggesting that *in vitro* propagation recapitulates native events fairly well.

Finally, it should be emphasized that although mutant PrP^Sc^ molecules were used in this study to uncover the dissociation between seeding ability and infectivity, the results are directly relevant to the mechanisms responsible for forming naturally occurring prions because normal wild type brain homogenates and normal wild type mice were used in sPMCA seeding assays and *in vivo* bioassays, respectively. The contrast between highly infectious wild type PrP^Sc^ and minimally infectious ΔPBD-PrP^Sc^ molecules provides a novel paradigm that can be used to determine the specific structural basis of prion infectivity.

## Supporting Information

Supporting Information S1 **Supporting methods and nine supporting figures: S1–S9.** This supporting information contains figures that are referenced in the main text and additional methods for the supporting figures.
(DOC)
