# Supplementary material for: Dissociation of Infectivity from Seeding Ability in Prions with Alternate Docking Mechanism
Source: PLoS Pathog. 2011 Jul 14;7(7):e1002128. doi: 10.1371/journal.ppat.1002128 (PMC3136465; doi:10.1371/journal.ppat.1002128)
Supplement: Supporting Information S1 — Supporting methods and nine supporting figures: S1–S9. This supporting information contains figures that are referenced in the main text and additional methods for the supporting figures. (DOC) [file ppat.1002128.s001.doc]

**Supporting Information S1**

**Supporting Methods**

**Solubility and phospholipase release assays of wild-type and mutant PrP^C^**

For solubility and PI-PLC release assays, lysis buffer contains 150mM NaCl, 50mM Tris pH 7.5, 0.5% Triton X-100, and 0.5% sodium deoxycholate (DOC). Assays were carried out as previously described [1].

**PrP^Sc^ purification for binding assay**

For assessing the PrP-binding behavior of purified PrP^Sc^, RML scrapie-infected brain homogenate was digested with 150 U/mL S7 micrococcal nuclease (Roche, Indianapolis, IN) in PBS, 1% Triton X-100, 2 mM CaCl_2_ for 30 min. at 37°C with end-over-end rotation. This was then treated with immobilized trypsin (Mag-Trypsin, Clontech, Mountain View, CA). 10 µL of 5% Mag-Trypsin suspension was rinsed in 200 µL ammonium bicarbonate pH 8.0, added to the sample, and incubated for 1 hr. at 37°C with end-over-end rotation. Mag-Trypsin was magnetically separated, and the sample was centrifuged at 100,000 x*g* for 1 hr. at 4°C. PrP^Sc^ was resuspended in PBS 1% Triton, vortexed for 15 s., agitated by pipetting, and sonicated at 85 power for 2 x 30 s. intervals. The preparation was then clarified of debris by centrifugation at 500 x*g* for 15 min.

**
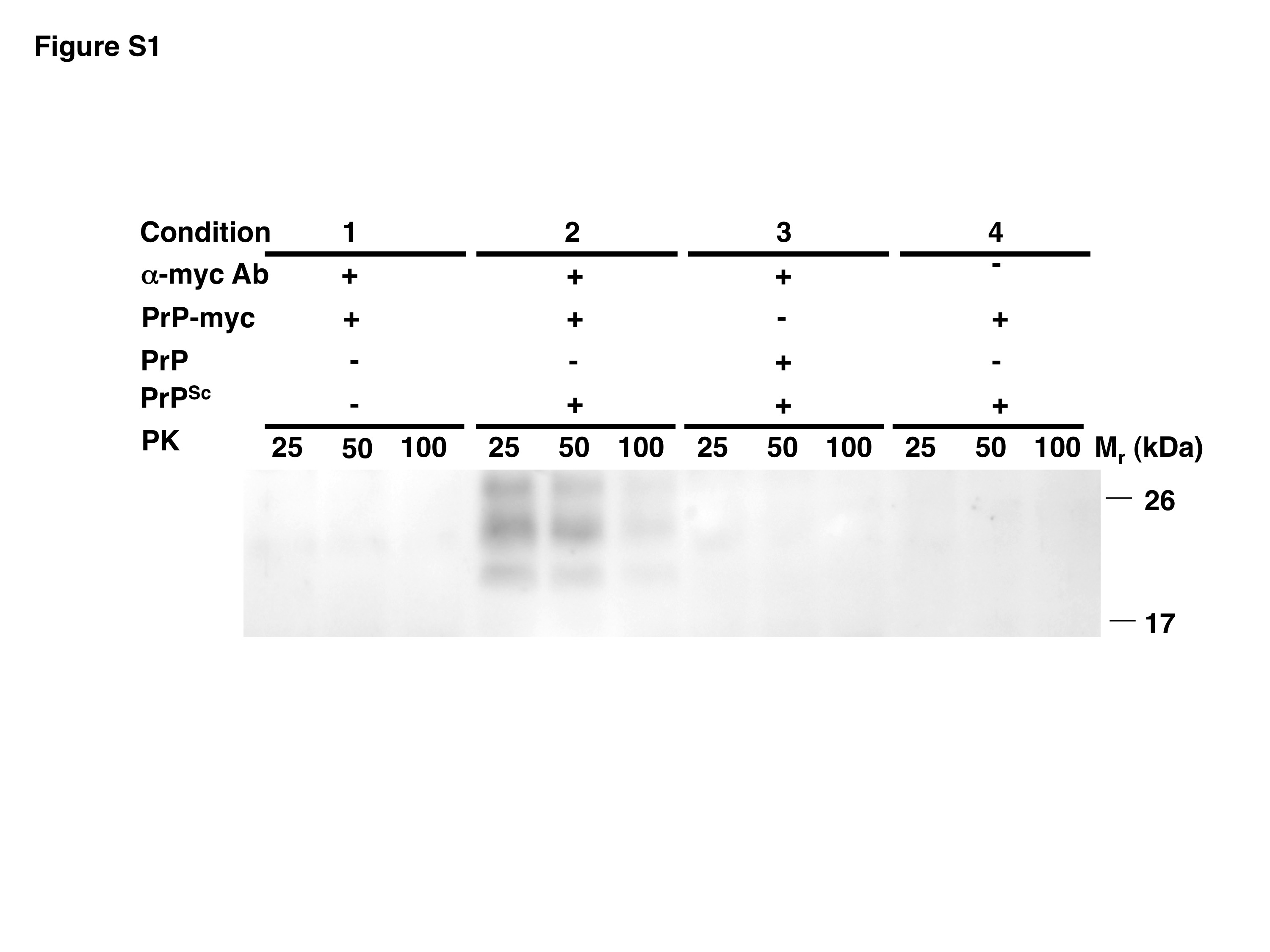
**

**Figure S1. Validation of myc-capture PrP^Sc^-PrP^C^ binding assay.** RML scrapie-infected mouse brain homogenate (PrP^Sc^) was incubated with wild-type myc-tagged PrP23-230 purified from *E. coli*. Bound PrP^Sc^ was captured with 9E10 anti-myc antibody on magnetic protein A Dynabeads (condition 2). Control reactions were performed, lacking PrP^Sc^ (condition 1), with untagged PrP substituted for PrP-myc (condition 3), or lacking the α-myc antibody (condition 4). Bound PrP^Sc^ was detected by 25, 50, or 100 μg/mL proteinase K digestion and anti-PrP (6D11) immunoblot.

**
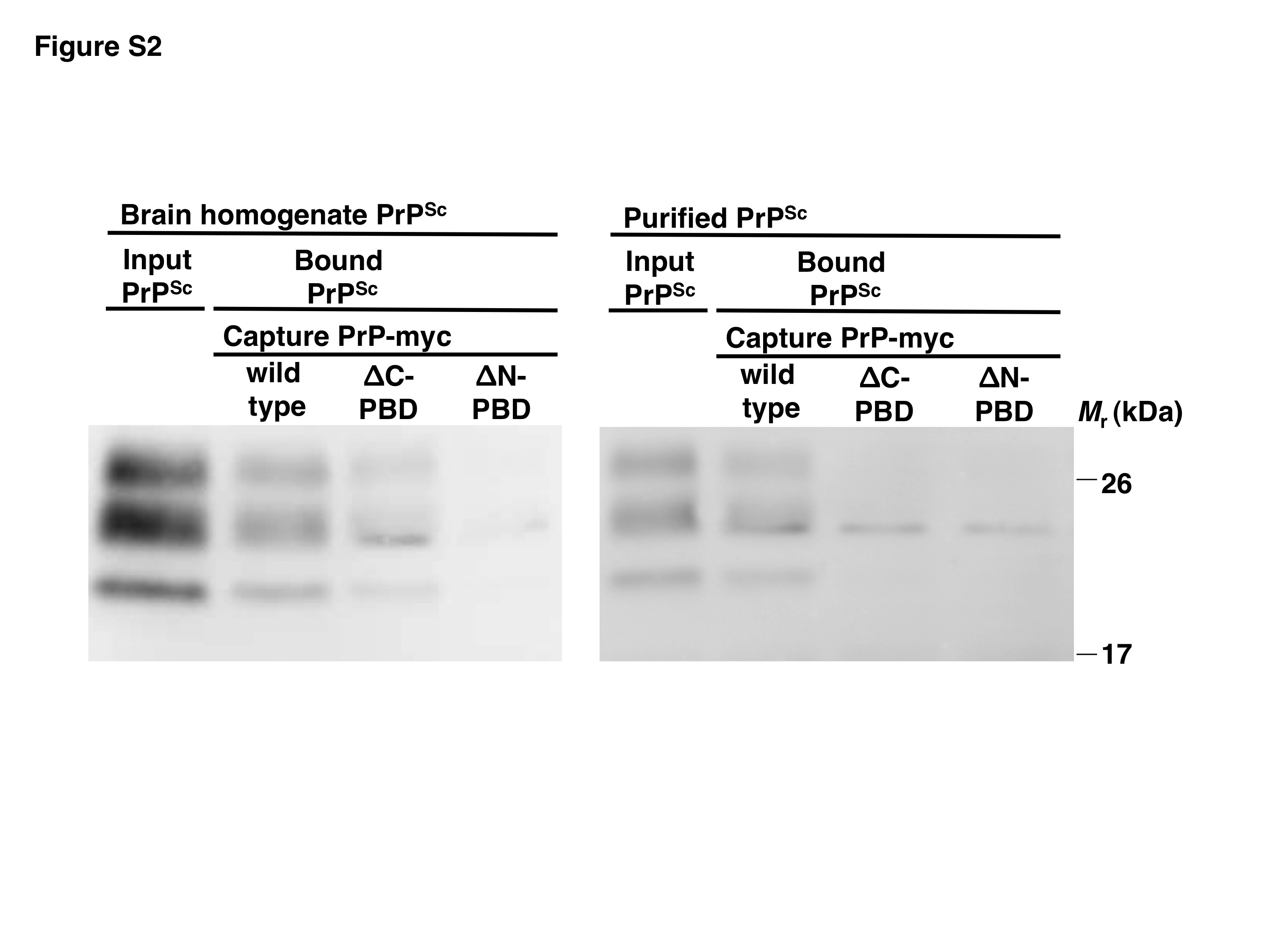
**

**Figure S2. Interaction of crude and purified PrP^Sc^ with PrP molecules.**  PrP^Sc^, from crude clarified RML scrapie-infected brain homogenate (*left*) or purified by nuclease digestion, protease digestion, detergent extraction, and ultracentrifugation (*right*), was incubated with purified myc-tagged PrP of wild-type sequence or lacking the central (ΔC-PBD: Δ100-109) or N-terminal (ΔN-PBD: Δ23-28) polybasic domain. Bound PrP^Sc^ was captured with 9E10 anti-myc antibody on magnetic protein A Dynabeads, and detected by 25 μg/mL proteinase K digestion and anti-PrP (6D11) immunoblot. The ~25 kDa signal in each lane represents incompletely digested cross-immunoreactive anti-myc IgG light chain.

**
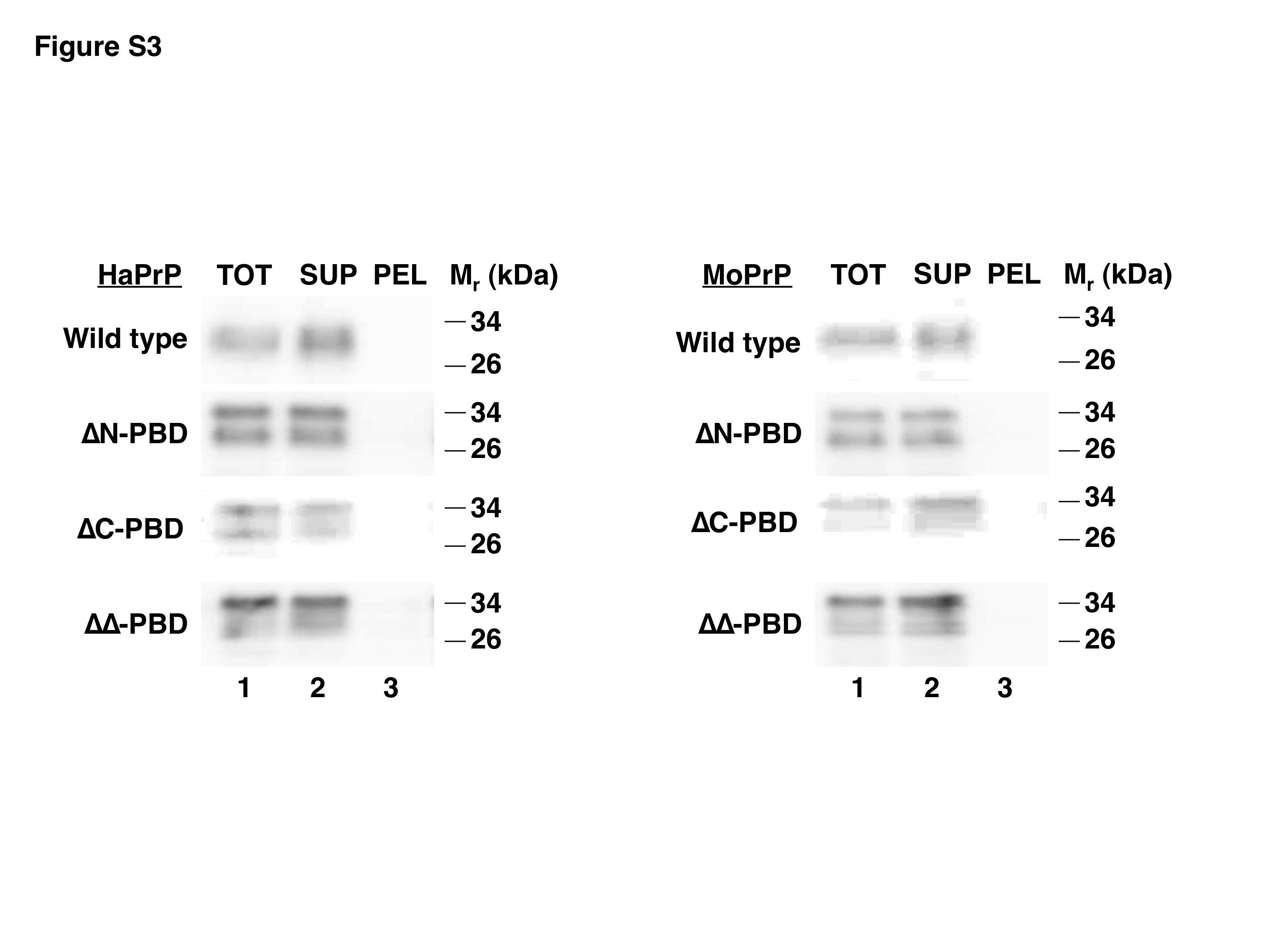
**

**Figure S3. Detergent solubility of PrP molecules expressed in CHO cells.**

CHO cell lines stably expressing recombinant wild-type and polybasic domain-mutant PrP were harvested in lysis buffer containing 150mM NaCl, 50mM Tris pH 7.5, 0.5% Triton-X 100, and 0.5% DOC. A portion of each cell lysate was removed, while the remainder was centrifuged at 100,000x*g*, and supernatant and pellet fractions were isolated. Equivalent amounts of the total lysate (*TOT, lane 1*), supernatant (*SUP*, *lane 2*), and pellet (*PEL*, *lane 3*) fractions were resuspended in SDS-PAGE loading buffer and PrP was detected in each sample by Western blotting with the antibody 27/33.

**
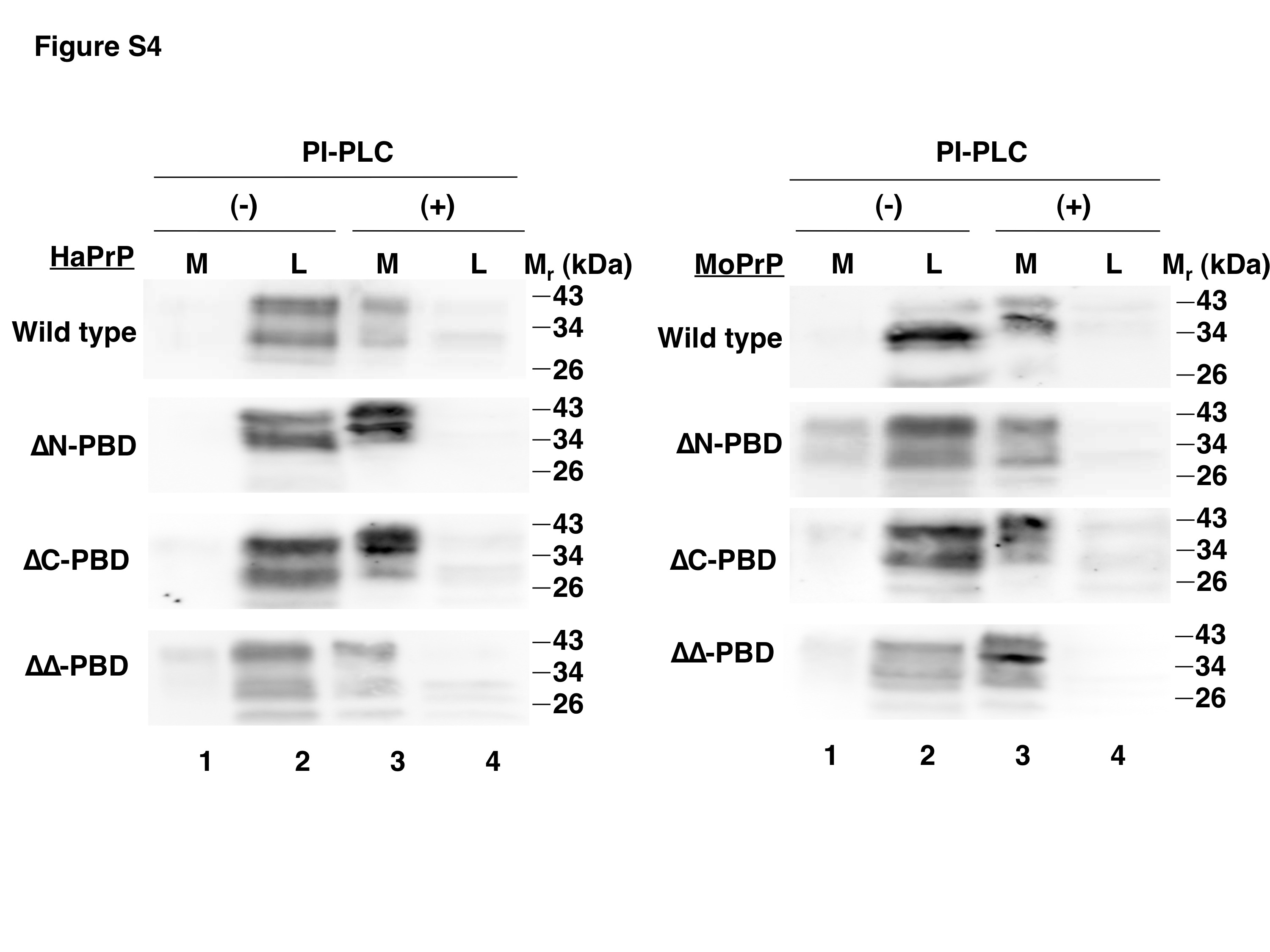
**

**Figure S4. Cell surface localization of PrP molecules expressed in CHO cells.**

CHO cell lines stably expressing wild-type or polybasic domain deletion mutant PrP were treated with (*lanes 3* and *4*) or without (*lanes 1* and *2*) phosphatidylinositol-specific phospholipase C (PI-PLC) to assess anchorage to the outer leaflet of the plasma membrane via the glycosylphosphatidylinositol (GPI) anchor. Following treatment, proteins in the PI-PLC incubation media (*M*, *lanes 1* and *3*) were precipitated, and cell lysates (*L*, *lanes 2* and *4*) were prepared in lysis buffer. PrP was detected by Western blotting with the antibody 27/33.


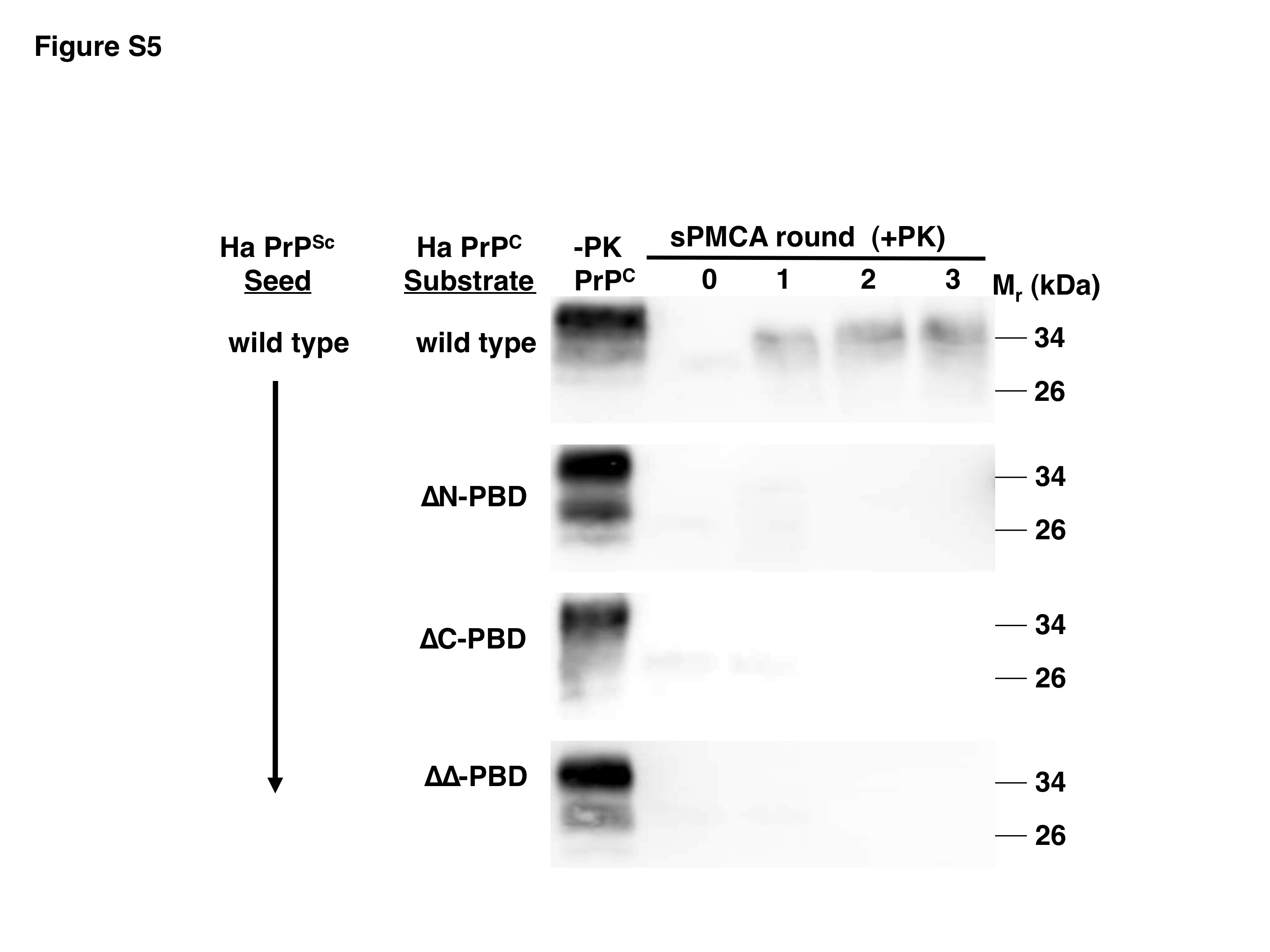


**Figure S5. Propagation of PrP^Sc^ using hamster PrP substrates.** Sc237 scrapie-infected hamster brain homogenate was propagated by sPMCA with wild-type or polybasic deletion mutant HaPrP^C^ prepared from Chinese hamster ovary (CHO) cells. Reactions were supplemented with synthetic poly(A) RNA, as described in methods. One sample of each reaction was not subjected to protease digestion (-PK). All others were subjected to limited proteolysis with 50 μg/mL proteinase K. PrP was detected with mAb 27/33.


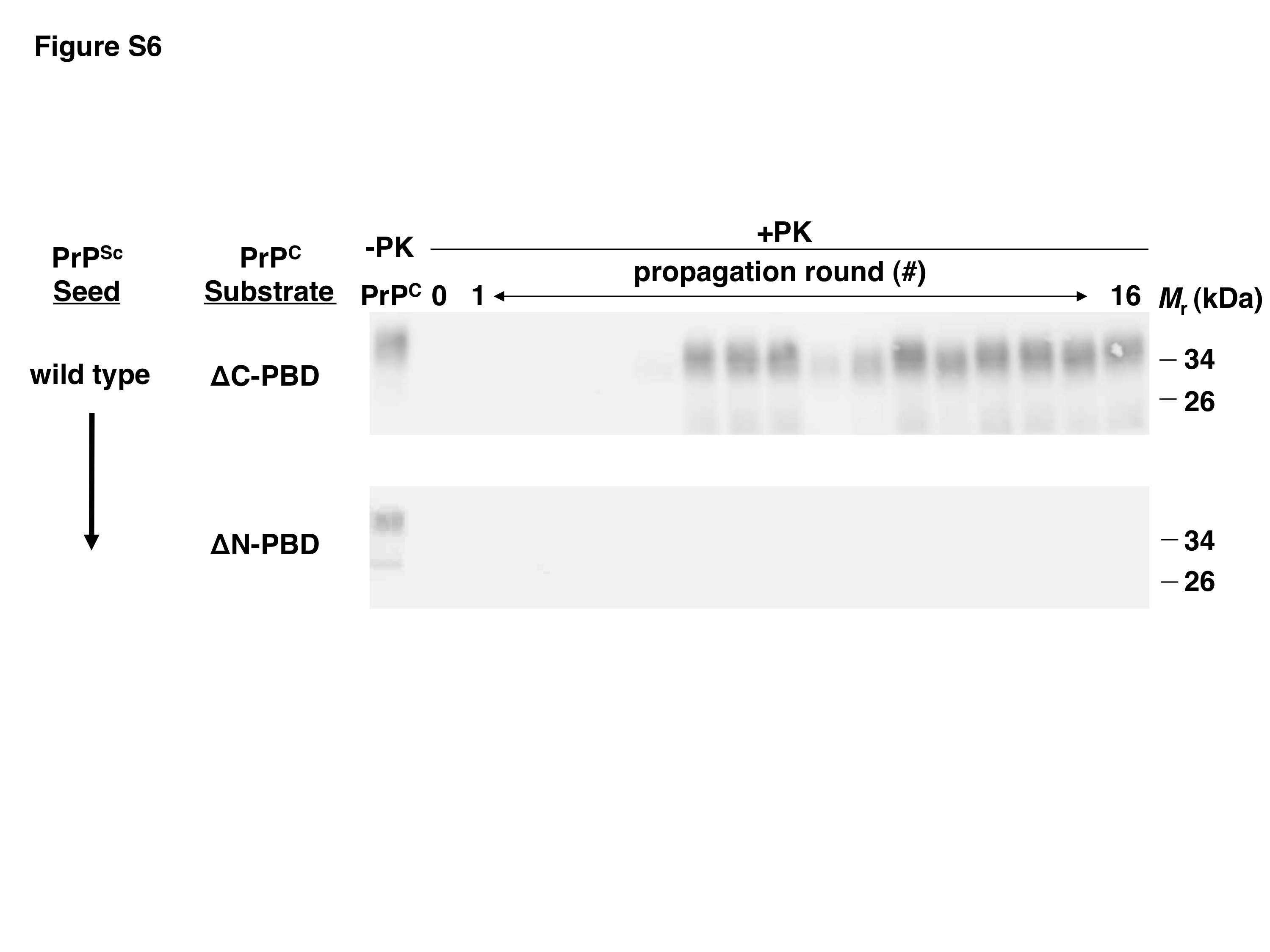


**Figure S6. Extended serial propagation of ΔC-PBD PrP^Sc^.** RML scrapie-infected mouse brain homogenate was propagated by sPMCA for sixteen rounds with ΔC-PBD or ΔN-PBD polybasic deletion mutant PrP prepared from Chinese hamster ovary (CHO) cells. Reactions were supplemented with *Prnp^0/0^* mouse brain homogenate. One sample of each reaction was not subjected to protease digestion (-PK). All others were subjected to limited proteolysis with 25 μg/mL proteinase K. PrP was detected by Western blot with mAb 27/33.

**
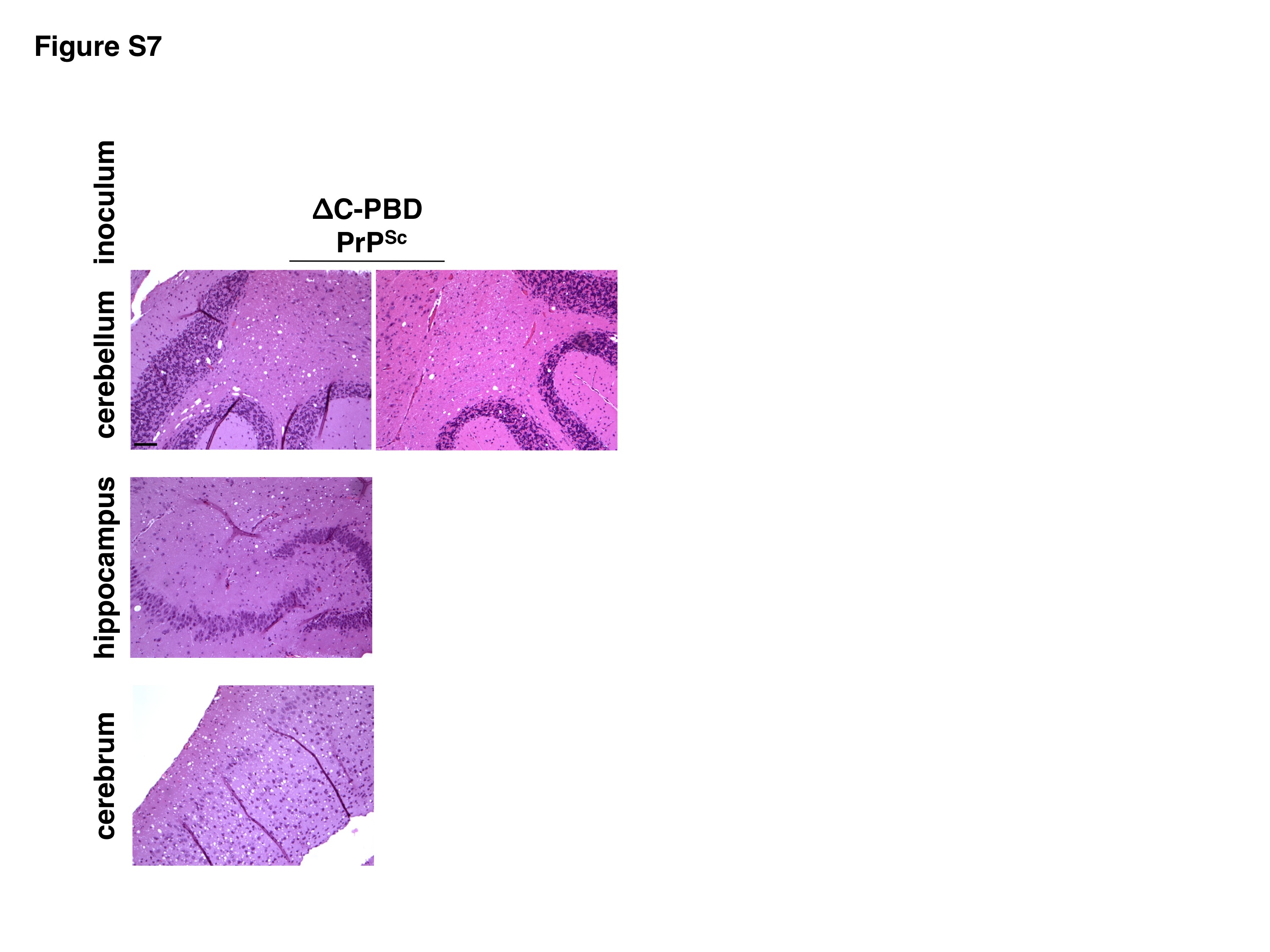
**

**Figure S7. Additional neuropathological analysis of mice inoculated with *in vitro*-generated ΔC-PrP^Sc^ molecules.** Brains were dissected from wild-type mice showing terminal scrapie signs. Histological sections were stained with hematoxylin and eosin (H&E), showing neuropathology of cerebellum, hippocampus, and cerebrum. The black bar denotes 100 μm.

**
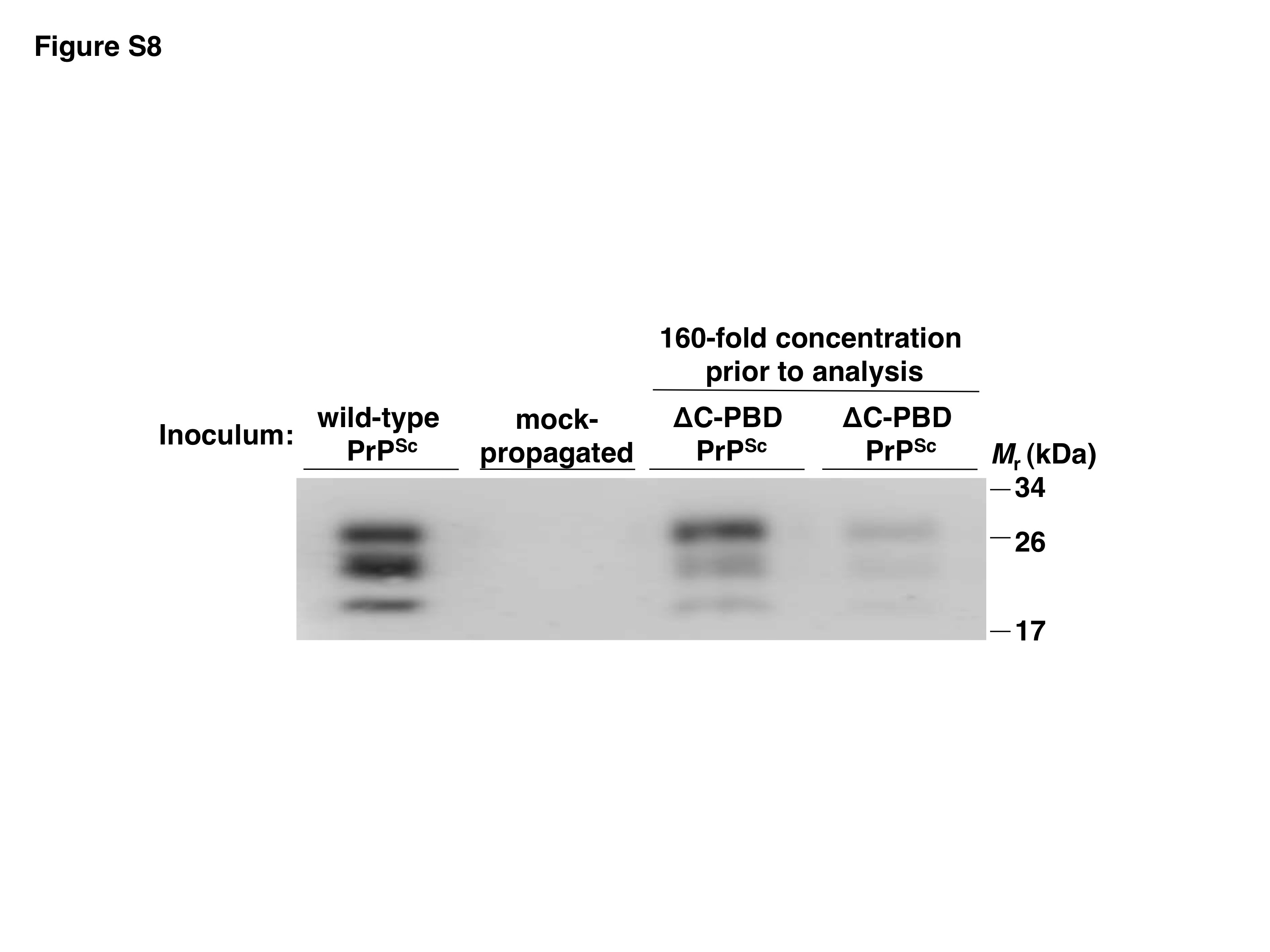
**

**Figure S8. Biochemical analysis of mice inoculated with *in vitro*-generated PrP^Sc^ molecules, adjusted for protease-resistant PrP.** Brains were dissected from wild-type mice showing terminal scrapie signs (one inoculated with wild-type PrP^Sc^ and two separate animals receiving ΔC-PBD PrP^Sc^ inocula) or similarly aged mice not displaying scrapie signs (mock-propagated). Brain homogenate was adjusted for levels of protease-resistant PrP by loading 160-fold more sample from brains of mice inoculated with ΔC-PBD PrP^Sc^. Each was treated with 25 μg/mL proteinase K and detected by anti-PrP (6D11) immunoblot.

**
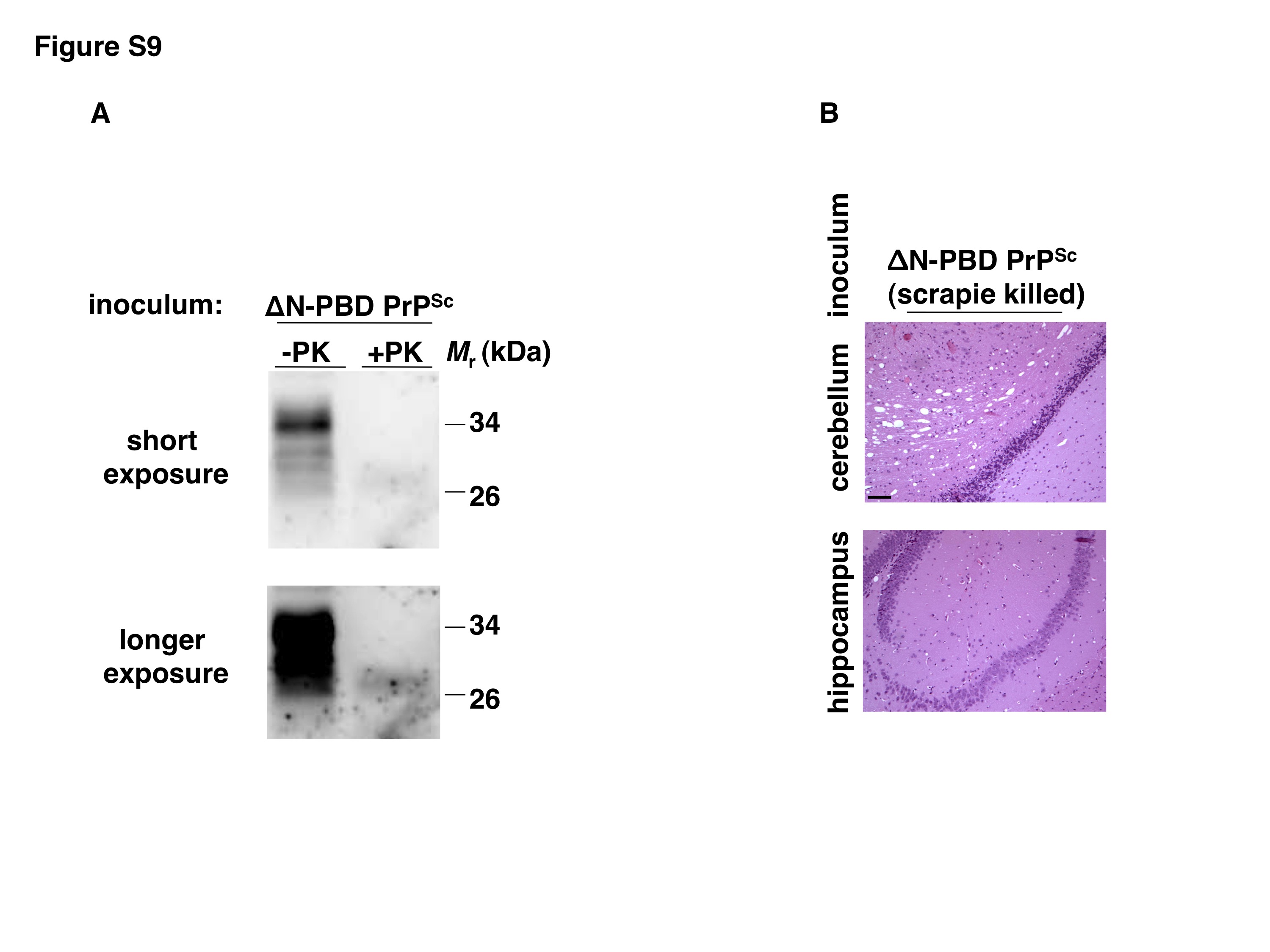
**

**Figure S9. Analysis of scrapie sick wild-type mouse inoculated with *in vitro*-generated ΔN-PrP^Sc^ molecules.** The brain was dissected from the single mouse showing terminal scrapie signs. (*A*) Equivalent amounts of 10% brain homogenate were treated with buffer (- PK) or 25 μg/mL proteinase K (+ PK to show PrP^Sc^) and detected by anti-PrP (6D11) immunoblot. Two exposures of the same blot are shown to illustrate the small amount of protease-resistant PrP. (*B*) Histological sections were stained with hematoxylin and eosin (H&E), showing neuropathology of cerebellum and hippocampus. The black bar denotes 100 μm.

**Reference**

1. Geoghegan JC, Miller MB, Kwak AH, Harris BT, Supattapone S (2009) Trans-dominant inhibition of prion propagation in vitro is not mediated by an accessory cofactor. PLoS Pathog 5: e1000535.
